# Gender differences in agricultural productivity: evidence from maize farm households in southern Ethiopia

**DOI:** 10.1007/s10708-019-10098-y

**Published:** 2019-11-04

**Authors:** Girma Gezimu Gebre, Hiroshi Isoda, Dil Bahadur Rahut, Yuichiro Amekawa, Hisako Nomura

**Affiliations:** 1 Department of Agricultural and Resource Economics, Graduate School of Bioresource and Bioenvironmental Sciences, Kyushu University, Fukuoka, Japan; 2 Department of Agricultural Economics, Aksum University, Aksum, Ethiopia; 3 Faculty of Agriculture, Kyushu University, Fukuoka, Japan; 4 International Maize and Wheat Improvement Center (CIMMYT), Socioeconomics Program, El Batan, Mexico; 5 College of International Relations, Ritsumeikan University, Kyoto, Japan; 6 Center for Promotion of International Education and Research, Faculty of Agriculture, Kyushu University, Fukuoka, Japan

**Keywords:** Gender difference, Maize productivity, Household head, Dawuro zone, Ethiopia

## Abstract

This study examines the impact of gender differences on maize productivity in Dawuro Zone, southern Ethiopia. Our study addressed the limitations of the previous studies in two ways. First, the study separately assessed gender differences in productivity between de facto female-headed households and de jure female-headed households and revealed that female-headed households are not homogenous. Second, the study separately examined the impacts of the covariates on male-headed households and female-headed households using an exogenous switching treatment effect model. We find the existence of gender differences in maize productivity between male-headed households and female-headed households. The maize productivity of male-headed households was overall 44.3% higher than that of female-headed households. However, if female-headed households received the same return on their resources as male-headed households, their productivity would increase by 42.3%. This suggests agricultural policy should target female-headed households to help reduce the productivity gap between male-headed households and female-headed households. Finally, the distributions of the gender differentials between male-headed households and female-headed households are more pronounced at mid-levels of productivity.

## Introduction

Agricultural productivity, in general, is low in many sub-Saharan African countries where most farmers are smallholders. It is even lower for female farmers, who comprise 50% of the agricultural labor force in the region (FAO 2011b). A report by the International Fund for Agricultural Development cited in FAO (2011b) showed that the percentages of female-headed households (FHHs) in rural eastern and southern Africa are ranges from 25 to 60%. Female-headed households are not homogenous. They can largely be divided into two categories: (a) households headed by women who are not married, are divorced or widowed (*de jure* FHHs) and (b) women whose spouses are away from home because of work or other reasons (*de facto* FHHs). The main reasons for the increase in the number of female-headed households are the migration of men away from rural areas to seek jobs elsewhere, widowhood, divorce, and other family disruptions (FAO 2011b; Kassie et al. 2014).

The extent of agricultural productivity differences between male and female farmers varies across and within countries in sub-Saharan Africa. Empirical evidence shows that the gender differences in agricultural productivity across sub-Saharan African countries are generally around 20 to 30%, with an average of 25% (FAO 2011b; Aguilar et al. 2014; Kilic et al. 2015; Mukasa and Salami 2015). The productivity difference between MHHs and FHHs from northern to southern Ethiopia ranges from 30 to 65%, respectively (Tiruneh et al. 2001; Aguilar et al. 2014; Challa and Mahendran 2015). One of the key reasons’ women farmers have lower productivity is the difference in the use of inputs such as improved seed, fertilizer, and labor, and access to other resources influencing productivity such as education, extension, and credit. However, it is important to note that *de facto* FHHs and *de jure* FHHs are not equally constrained. Indeed, FAO (2010) notes that, while the *de facto* FHHs who receive remittance from their husbands can mitigate the effects of the absence of male agricultural laborers by increasing investment in farm tools and inputs, remittance does not procure labor itself

The vast majority of the recent literature and a review of early studies undertaken by Quisumbing (1996) confirm that estimates of the gender productivity gap become insignificant taking into account the difference in use of inputs, access to productive resources, and characteristics of individual farmers. Following recent empirical reviews, FAO (2011b) takes this further: “If women farmers used the same level of the resource as men on the land they farm, their land productivity could increase by 20–30%. This could raise the total agricultural output in developing countries between 2.5 and 4%, which could, in turn, reduce the number of undernourished people in the world by 12–17%” (pp 5).

As Ethiopia is multicultural, women’s roles in agriculture vary across regions. Nonetheless, women from every region face gender-specific constraints related to socio-cultural forces that serve to reduce their agricultural productivity and to limit their ability to ensure production. For example, women in southern Ethiopia face a serious gender gap in access to productive resources, as farming there is culturally considered a man’s task (Aguilar et al. 2014).

Previous studies on gender and agriculture tend to focus on understanding efficiency and productivity differences between MHHs and FHHs (e.g., Tiruneh et al. 2001; Njuki et al. 2006; Thapa 2008; Ragasa et al. 2012; Challa and Mahendran 2015). There has been little or no attention paid to gender differences between *de facto* FHHs and *de jure* FHHs. Moreover, these studies measure gender effects on productivity by applying pooled regression with a binary gender variable. This type of analysis alone does not generate sufficient evidence to draw understanding for policy options related to gender and agricultural productivity. This is because it fails to recognize the interaction between gender and other covariates in the model. It can only provide the intercept effect (i.e. results in a parallel shift up or down to various productivity profiles).

This paper examines the productivity differences between MHHs and FHHs, including analyses that comparatively examine *de facto* FHHs and *de jure* FHHs in maize-growing areas of Ethiopia. This is done by taking into account observable and unobservable factors. Specifically, it aims to assess access to productive resources by different gender stakeholders; to analyze the factors that contribute to gender differences in productivity; to evaluate the measure of efficiency in the use of resources for production; and to measure and compare the productivity gap between MHHs and FHHs. In doing so, the paper shines a light on the literature on gender and agriculture, as well as the literature on gender and development, in Ethiopia and elsewhere in the developing world.

The rest of this paper is organized as follows: "Literature review" section provides a literature review, "Materials and methods" section presents materials and methods, "Results and discussion" section provides findings and discussion, and "Conclusion and policy implications" section concludes the paper.

## Literature review

### Measuring gender differences in agricultural productivity

A substantial body of the existing studies indicate that gender inequality in access to productive resources such as land, improved varieties, fertilizers, farm equipment, labor, training, and information lead to the difference in agricultural productivity between male and female farm households. However, the extent of the differences and the relative importance of their potential drivers depend on the country or region, the sample size, the type of crop, the unit of measurement, or the method of analysis (Table 1). To measure the magnitude of agricultural productivity differentials and unpack their potential sources, the commonly-used approaches by existing studies are production function estimates (e.g. Saito et al. 1994; Udry et al. 1995; Tiruneh et al. 2001; Njuki et al. 2006; Ragasa et al. 2012; Challa and Mahendran 2015) and Blinder-Oaxaca decomposition methods (e.g. Aguilar et al. 2014; Kilic et al. 2015; Oseni et al. 2015; Mukasa and Salami 2015; Slavchevska 2015; Ali et al. 2016). Production function estimates measure the differentials by coefficients while the decomposition methods measure them by covariates and its coefficients. Moreover, some studies used switching regression in a counterfactual manner to measure production efficiency (e.g., Solis et al. 2007; Ahmed 2012) and test whether the difference is driven by the differences in observable characteristics or return to these characteristics.

**Table 1:** Summary of literature review on gender difference in agricultural productivity

Author/s and year	Country studied	Crop	Sample	Dependent variable	Gender indicator	Productivity gap	Main reasons for the gap
Saito et al. (1994)	Kenya	Main crops	750 households	Value of output/ha	Head dummy	8.4%	Difference in input use
Udry et al. (1995)	Burkina Faso	All crops, sorghum, and vegetables	150 households farming 4655 plots in four years	Yield/ha	Farmer dummy	18% for all crops, 40% for sorghum and 20% for vegetables	Lower use of productive inputs by women
Tiruneh et al. (2001)	Ethiopia	All crops	180 households	Value of output/ha	Head dummy	35%	Women’s lower levels of inputs and limited access to extension services
Njuki et al. (2006)	Kenya	Crops, tree and cattle	40 farmers	Value of output/ha	Plot manager dummy	No significant difference	Age, female labor and land size have a significant effect
Goldstein and Udry (2008)	Ghana	Maize and Cassava	60 married couples with each four villages	Profit	Farmer dummy	Wives achieve lower profit than husband	Women’s land tenure insecurity leads to less investment in land fertility
Akresh (2008)	Burkina Faso	All crops	2406 households	Value of output/ha	Farmer dummy	32% (ranges from 32% to 50%)	Difference in farm size and vulnerability to rainfall shocks
Alene et al. (2008)	Kenya	Maize	800 households	Yield/ha	Head dummy	23%	Women’s lower access to land and education
Kinkingninhoun-Meˆdagbe et al. (2010)	Benin	Rice	145 farmers	Yield/ha	Farmer dummy	27%	Women lack timely access to services for farming and transportation
Peterman et al. (2011)	Nigeria and Uganda	All major crops	Nigeria: 3706 households; Uganda: 3625 plots	Value of output/ha	Head and plot manager dummy	Nigeria: 75% Uganda: 28.6%	Differences in socio-economic characteristics, farm input, and crop choice
Ragasa et al. (2012)	Ethiopia	All major crops	7530 households and 31,450 plots	Value of output/ha	Head and plot manager dummy	Women achieve lower productivity	Women’s lower access to quality extension services, price information, and other inputs
Aguilar et al. (2014)	Ethiopia	All major crops	1518 households	Value of output/ha	Plot manager dummy	Average: 23.4% (Tigray: 33.1%, South: 61.4%)	Differences in access to productive resources and its returns
Kilic et al. (2015)	Malawi	All major crops	16,372 plots	Value of output/ha	Plot manager dummy	25%	Differences in productive resources/endowments
Challa and Mahendran (2015)	Ethiopia	Coffee, maize and bean	150 households	Value of output/ha	Head dummy	66.76%	Women’s lower access to productive resources/inputs
Backiny-Yetna and McGee (2015)	Niger	All crops	3968 households	Value of output/ha	Plot manager dummy	Average: 19% but ranges up to 66%	Shortage of male labor, fertilizer, and land owned
Mukasa and Salami (2015)	Nigeria, Tanzania and Uganda	All major crops	Nigeria: 4017 Tanzania: 2530, Uganda: 2029 plots	Value of output/acre	Plot manager dummy	Nigeria: 18.6% Tanzania: 27.4%, and Uganda: 30.6%	Difference in endowment and structural disadvantages of women’s land size, land quality, labor inputs, and household characteristics
Oseni et al. (2015)	Nigeria (North and South)	All major crops	2431 households farming 4240 plots	Value of output/ha	Sex of plot manager	North:28% South: no significant difference	Structural disadvantages of women in the northern parts
Slavchevska (2015)	Tanzania	All major crops	6945 plots from 2182 households	Value of output/ha	Sex of plot manager	Weak gender difference	Differences in size of plot area and family labor
Ali et al. (2016)	Uganda	All major crops	6999 plots	Value of output/acre	Sex of plot manager	17.5%	Difference in child dependency ratio, access to transportation, improved seed, and pesticide use

*Source*: Compilation of authors from various sources

Concerning the measurement of agricultural productivity using the sex dummy, the commonly-used approaches by existing studies consider the sex of the household head, the sex of the farmer, and the sex of the plot manager or decision maker (Table 1). The headship is the most widely-used approach by many studies; however, it does not take into account the contribution of women in a male-headed household and men in a female-headed household (Doss 2018). The approach to using the sex of the farmer (or the plot manager) may be adopted to measure the productivity difference between men and women within the same household; however, this approach is also problematic if farmers are misidentified in the household.^1^ In some cultural contexts, women are considered helpers for men working on the farm, but not as farmers because they spend more time working in the homestead. This situation holds particularly true in the Dawuro Zone, southern Ethiopia. Indeed, Ragasa et al. (2012) noted that extension services in Ethiopia do not always consider women to be farmers. This kind of study is usually completed in western Africa, where men and women often manage separate plots; meanwhile, this practice is much less common in eastern Africa. Even studies based on the sex of the plot managers in the east African region show a significant overlap along gender lines. For instance, in their study, de la O Campos et al. (2016) used nationally-representative data from Uganda and found that 92% of female-held plots and 77% of female-managed plots belong to female-headed households.

In addition, in southern Ethiopia, particularly in the Dawuro Zone, household headship is closely related to the occupational status as farmer because land entitlement always belongs to the heads. Who becomes a household head is then a matter of concern.^2^ In this regard, the existing social norms permit significant gender biases in favor of men. Men typically inherit property rights of land and other household assets of the household, which leads them to become the household head. Women are normally supposed to be a household head only in the absence of their male counterparts, whether due to the death of their husband, divorce, a husband’s seasonal migration for wage work, or disappearance. Women in male-headed households typically have no separate plot for themselves while women in female-headed households own plots provided by their husband or inherited from their husband in accordance with the culture and traditions of the Dawuro community. Considering these circumstances this study uses the sex of the headship to compare gender differences in maize productivity.

Regarding the unit of productivity measurement, the yield (quantity of output per unit area) and the value of output per unit of land are the most commonly-used approach by the existing studies (Table 1). Yield measurement is simple and works best when farmers grow a single crop on a plot; however, it is less straightforward when farmers grow multiple crops on a single plot of land at the same time (Saito et al. 1994). When one is interested in evaluating the productivity of a single crop across different periods, using the yield method is problematic for the plots where multiple crops are grown across different seasons. Even for a single production season, it is difficult to measure the yield of crops that are continuously harvested (Doss 2018). In Ethiopia, maize-growing households harvest some portion of their maize in the course of the growing season (at the green stage, particularly for their family consumption). The majority is harvested at the peak maturity (grain) stage. In addition, some farm households apply intercropping practices on their maize plots. These factors raise complexities for yield analysis.

Previous studies commonly use the value of output (summing the value of individual crops) as a measure of farm productivity, especially when more than one crop is grown on a plot (Saito et al. 1994). Doss (2018) notes that if one is interested in measuring the productivity of individual crops on the intercropped plots, he/she can allocate a unit area of land for the crop grown to its respective output. For example, if a hectare of the plot is intercropped equally with maize and legumes, then maize is considered to be grown on half a hectare and legumes on another half a hectare, with the quantity of the output per hectare of each crop calculated accordingly.

Using the market value of output as the measure of productivity is conceptually clearer and solves the limitations of the yield method; however, it is also problematic if a given quantity of outputs of the same product receives different prices across different seasons or between different village markets. The common approach to compute the value of each crop applied by the existing studies is to use village-level median prices based on farm household self-reported sales information. Table 1 provides a summary of extant studies on gender productivity differences conducted in sub-Saharan African countries.

The existing studies used different units to gauge the contribution of different factors impacting gender differences in agricultural productivity. Among those factors, the most difficult to measure in the context of developing countries is labor, which is the most important input in the production process. Because smallholder farms in developing countries typically employ family labor, there is no wage income or written records of labor time to estimate the family labor input (Arthi et al. 2016). This situation is particularly applicable in Ethiopia. In most of the previous studies, farmers were asked to recall the amount of labor used for their plot for the previous farming season (Doss 2018). This leads to biases in reporting on labor time and affects the quality of data collected in the developing country (Arthi et al. 2016). The farming activities are not as regular as other office works, and many of these activities are carried out jointly with other household activities (Doss 2018). The seminal work by Arthi et al. (2016) points out that the biases in farm labor data are derived from reports on the weeks and days worked, not from the hours worked per day. This is because some farmers or family members will work longer hours per day than some other farmers or family members. As a result, a day laborer could have a different marginal contribution to farm output (Doss 2018). Since male and female labor is not substitutive in agriculture (Doss 1999), most of the existing studies have separately estimated the labor inputs of men and women. Some of these studies (e.g., Saito et al. 1994; Tiruneh et al. 2001; Njuki et al. 2006; Challa and Mahendran 2015) estimated the marginal productivity of family labor using shadow wage rates, which represent the opportunity cost of the family labor time (Sharma 2013).

### Maize production and productivity in Ethiopia and the global context

Maize is the most widely grown crop in Ethiopia, and both men and women make significant contributions to maize farming in Ethiopia. Among cereals, maize accounts for the largest share of total crop production in the country. Between 2007 and 2017, the land area covered by maize increased from 1.69 to 2.14 million hectares. During the same period, the average national yield was about 2.6 tons per hectare, with minimum and maximum yields of 1.2 and 3.67 tons per hectare in 2016 and 2017, respectively. In the meantime, the number of smallholder farmers involved in maize production increased from 7.5 to 10.8 million. Averaged over the period from 2007 to 2017, maize was grown on 21.1% of the total area for cereal crop cultivation in Ethiopia by 9.5 million smallholder farmers. During that period, those smallholders produced a yearly average of 6.27 million tons of maize, which accounted for over 30% of the total cereal production (CSA 2015, 2016, 2017).

In the global context, Ethiopia is the fourth largest maize producer in Africa (Demeke 2012) and the second largest producer in eastern and southern Africa, following South Africa (FAO 2017, FAOstat). Moreover, Ethiopia has a higher average maize productivity compared to the continental average of Africa, although it is still lower than the world average and much lower than that of developed counties.

### Maize varieties in Ethiopia

In Ethiopia, there are two categories of maize varieties under production: improved and local varieties. The improved maize varieties are classified into hybrids and open-pollinated varieties (OPVs). Hybrids have the highest yield but are more costly to adopt as the restoration of hybrid vigor requires the purchase of new seeds in each cropping season. OPVs generally have a lower yield than hybrids, but OPV seeds cost less than hybrids and can be recycled for up to three seasons without a significant yield loss (CGIAR 2014). In 2013, more than 16 hybrids and four OPVs were under production in Ethiopia. Out of them, hybrids accounted for 97%, while OPVs represented only 3% of Ethiopia’s total maize seed market. Among the existing improved varieties, BH660, BH540, and Pioneer hybrids dominate Ethiopia’s seed market (Abate et al. 2015). The major maize varieties used for production in Dawuro Zone are BH660, BH540, Pioneer hybrids, and local (recycled hybrids) varieties (Gebre et al. 2019). Thus, this study included only these hybrids and local varieties.

## Materials and methods

### Study area, data, and sampling

**Fig. 1:**
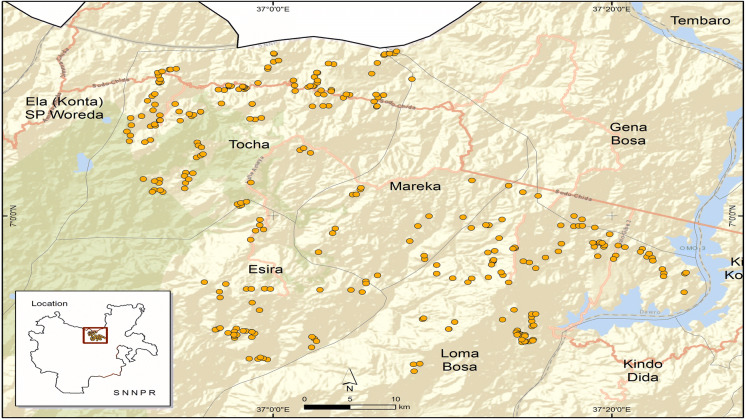
Map of the study area (Dawuro Zone) in southern Ethiopia Source: Author’s sketch using GPS data (2018)

Ethiopia is divided into eleven regional states. Each regional state is subdivided into zones, *woredas* (districts) and *kebeles* administrations.^3^ The South Nations, Nationalities, and People (SNNP) regional state is one of the largest regional states in Ethiopia. Dawuro Zone, located in the SNNP regional state, is in the major agricultural production area in the country. Before 2019, the Dawuro Zone consisted of five *woredas,* (Fig. 1) and one city administration. However, starting in 2019, the five *woredas* were divided into 10 *woredas.*^4^

The landscape of Dawuro is mostly mountains, plateaus, deep gorges, and lowland plains. The altitude of Dawuro ranges from 500 to 3000 m above sea level; thus, Dawuro exhibits climatic variations from lowland to highland. The climatic variations have enriched Dawuro with a variety of tree species and natural vegetation/forest. The majority of the Dawuro people (91%) live in rural areas (Negashi 2019), and their livelihood is based on a mixed crop-livestock production system. The principal crops produced in the Dawuro Zone include *ensete,* teff, maize, wheat, sorghum, barley, millet, coffee, beans, peas, spices, vegetables, and fruits. Though Dawuro has ample potential for agricultural production, its farm productivity is very low because farmers use traditional means of production. Dependence on natural rainfall coupled with poor market access makes the livelihood of farming households extremely stagnant (Abebe 2014). Both male and female-headed households are involved in agricultural activities in the Dawuro Zone; however, female-headed households are particularly vulnerable to poverty and food insecurity due to a lack of access to farmland, a shortage of farm labor, or not having draft animals for crop production. In some cases, young men (most often the husband) from male-headed households migrate to distant area in search of better incomes or wage labor. While the men are gone, women, or the wives, take all household food production responsibilities. A woman is also responsible for home activities, including childcare. These households are treated as *de facto* female-headed households in this study. This study is based on household and maize production data collected in the Dawuro Zone of the SNNP regional state in Ethiopia from April to June 2018.

Multi-stage purposive sampling techniques based on probability proportional to size were used to select districts, *kebeles*, and households in the Dawuro Zone. In the first stage, four districts named Loma (including Zisa), Mareka, Esara, and Tocha (Kachi and Tarcha zuria) were selected based on their maize production potential. In the second stage, six to eight *kebeles* growing maize were selected from each district. In the third stage, MHHs and FHHs were selected for the survey based on whether they farmed maize in the 2017/2018 production season.^5^ This was done with the assistance of agricultural development (DA) agents who keep in constant contact with the farm households in each *kebele.*^6^ Twenty maize farm households consisting of FHHs and MHHs were selected from each *kebele*. Accordingly, a sample of 560 (409 MHHs and 151 FHHs) smallholder maize producers was obtained for the survey. Out of the 151 female household heads, 89 and 62 were *de jure* and *de facto* FHHs, respectively.

In both male and female-headed households, the maize farming is based on a family farming system.^7^ The person most responsible for farming in the household was interviewed by using a semi-structured questionnaire. Aside from headship, other male and female family members were separately asked about their labor contribution (number of weeks worked, average number of days worked per week, and average number of hours worked per day) on maize plots, differentiated by activities such as land preparation, planting, weeding, harvesting, collecting, and so on.^8^ The household members were also asked about their labor time spent growing other crops and non-agricultural or household activities during the maize growing season. Subsequently, they were asked if any of their family members were not involved in their maize farming activities. Because of the social norms and physical requirements of farming in the Dawuro Zone, the amount of labor provided by male and female family members varies across different farming activities. Land preparation and planting are primarily considered a male task while women share other farming activities with men, such as weeding, harvesting, and collecting, in addition to their homestead activities such as caring for children, preparing food, and fetching water.

The collected data included detailed information on maize farming activities, inputs used and their prices, output and its prices as well as other socio-economic and demographic characteristics of the farm households. For the intercropped maize plots, the proportion was counted and appropriately treated to avoid biases in productivity measurement as suggested by Doss (2018). The detailed information on the amount of maize harvested in the course of growth (green) and grain stages was also collected to compute the total amount of harvest per hectare. The adult family members living in all the sampled households were involved in maize farming activities; however, the amount of time worked varied across activities. As male and female labor is not substitutive for each other in production (Doss 1999), the average number of hours worked on maize plots per day by male and female family members were separately counted. Finally, the total number of hours worked on maize plots by men is distributed equally among male family laborers. Likewise, the total number of hours worked by women is distributed equally among female family laborers. This is because the returns on labor hours in farming will vary across different activities (Doss 2018). The maize farmgate price data were collected from individual households to compute the total value of their maize output. However, in the study area, there is some variation in the maize output price across villages depending on farm distance to the market. Thus, the village level median prices based on farm household self-reported sales information were used to estimate the value of maize output. The median price is computed based on maize sales information from 10 observations within each *kebele*.

### Methods: empirical framework

To estimate the effects of gender on maize productivity, an exogenous switching treatment effect regression (ESTER) model in a counterfactual framework for measuring production efficiency was used. Two issues arise when we estimate the effects of gender on maize productivity. The first issue concerns the assumption that the social position related to gender status is endogenous to productivity. Some unobserved characteristics that influence gender could also influence productivity. The second issue is that an identical factor of production may have different effects between MHHs and FHHs on maize productivity. Thus, applying a pooled regression with a binary gender variable is not appropriate to estimate the causal effect of gender on maize productivity. The separate function for both MHHs and FHHs should be examined. In doing so, we take two-stage estimation procedures, which are shown as follows:

### Cobb–Douglas production function

The Cobb–Douglas production function is applied, in the first stage, to determine the relationship between the productivity level of the households and explanatory variables. Taking logarithms on both sides, the standard Cobb–Douglas production function for plot *i*, in household *j*, is specified as:

1 $$\begin{aligned} \ln Y_{ij} & = \beta_{0} + \beta_{1} \ln L_{ij} + \beta_{2} \ln T_{ij} + \beta_{3} Age_{j} + \beta_{4} Educ_{j} + \beta_{5} Cr_{j} + \beta_{6} Ext_{j} + \beta_{7} Dismarket_{j} \\ & \quad + \beta_{8} Rotate_{ij} + \beta_{9} Intercrop_{ij} + \beta_{10} Agroeco_{ij} + \beta_{11} Genderj + \varepsilon ij \\ \end{aligned}$$

where *Y*_*ij*_ is a measure of productivity for plot *i,* in *j* household; *L*_*ij*_ is labor input used in plot *i*, in *j* household; *T*_*ij*_ is a vector of land, capital, and other conventional inputs (such as maize seed and fertilizer) used in plot *i*, in *j* household; *Age*_*j*_ is the age of the household head; *Educ*_*j*_ is the educational level of the household head; *Cr*_*j*_ is indicators of access to credit service; *Ext*_*j*_ is an index of extension contact; *Dismarket*_*j*_ is plot distance from market; *Rotate*_*ij*_ is crop rotation practice on plot *i*, in household *j*; *Intercrop*_*ij*_ is intercropping practice on plot *i*, in household *j*; *Agroeco*_*ij*_ is a dummy variable representing agro-ecology of plot *i*,in household *j*; *Gender*_*j*_ is a dummy variable for the sex of the household head; ε_ij_ is the error term for plot *i*, in *j* household; and β’s are coefficients for constant terms and covariates included in the model. The dependent variable in the function is Total Value Product (TVP) per hectare,^9^ which is obtained by calculating the value of the maize yield using the village-level median price.

#### Marginal value product: measuring resource use efficiency

The Cobb–Douglas production function approach focuses on technical rather than allocative efficiency, which takes into account the allocation of resources among household members. To examine allocative efficiency, the Marginal Value Product (MVP) for the respective factors or inputs was calculated for both MHHs and FHHs. The function is specified as:

2 $$MVP = \beta_{i} AVP = \frac{Yi}{Xi}$$

where *β*_*i*_ is coefficient from Cobb–Douglass production function, *AVP* is average value product, *Y* is total value product, and *Xi* is the respective input $i$.

#### Exogenous switching treatment effect regression

In the second stage, the exogenous switching treatment effect regression (ESTER) model is used to estimate production functions for both MHHs and FHHs. We assume a Cobb–Douglas production function in estimating the switching regression model:

3 $$\left\{ {\begin{array}{*{20}l} {y_{m} = x_{m} \beta_{m} + \mu_{m} } \hfill & {if\;g = 1} \hfill \\ {y_{f} = x_{f} \beta_{f} + \mu_{f} } \hfill & {if\;g = 0} \hfill \\ \end{array} } \right.$$

Where subscript *m* and *f* indicate MHHs and FHHs, respectively. The variable *y* is referred to productivity outcome for each category of households, depending on the subscripts. A dummy variable, *g*, sets 1 for MMHs and 0 for FHHs; *x* is the vector of explanatory variables that determine maize productivity; *β* is the coefficient expressing how MHHs and FHHs respond to explanatory variables; and *μ* is the error term with zero mean and constant variance.

##### Conditional expectations, treatment, and heterogeneity effects

Equation (3) may not allow us to directly examine the role of gender in maize productivity for both MHHs and FHHs. We address this issue by estimating the counterfactual mean productivity of each group regarding how much the maize productivity of female-headed households would have been if the returns on their characteristics had been the same as the returns on the male-headed households’ characteristics, and vice versa. Following Carter and Milon (2005) and Kassie et al. (2014), we estimate the actual and counterfactual expected maize productivity of each category of the households as:

3a $$E(y_{m} |G = 1) = x_{m} \beta_{m}$$

3b $$E(y_{f} |G = 0) = x_{f} \beta_{f}$$

3c $$E(y_{f} |G = 1) = x_{m} \beta_{f}$$

3d $$E(y_{m} |G = 0) = x_{f} \beta_{m}$$

where *E* is the mean outcome of the expectation operator. Equations (3a) and (3b) represent the average maize productivity for MHHs and FHHs that are actually observed in the sampled households. Equations (3c) and (3d) are their respective counterfactual for expected average productivity. The use of the above conditional expectations, combined with the consideration of gender as a treatment variable, allows us to gauge the effects of gender on maize productivity. These conditional expectations for the above four outcome variables are presented in Table 2.

**Table 2:** Conditional expectations of productivity, treatment, and heterogeneity effects

Household types	MHHs	FHHs	Treatment effects
MHHs	a) $E\left( {y_{m} } \right.\left| {G = \left. 1 \right)} \right.$	c) $E\left( {y_{f} \left| {G = \left. 1 \right)} \right.} \right.$	$MHHsP$ = (a − c)
FHHs	d) $E\left( {y_{m} \left| {G = \left. 0 \right)} \right.} \right.$	b) $E\left( {y_{f} \left| {G = \left. 0 \right)} \right.} \right.$	$FHHsP$ = (d − b)
Heterogeneity effect (difference caused by unobserved characteristics)	$BH_{m}$ = (a − d)	$BH_{f}$ = (c – b)	

Cells a) and b) denote the average productivity that is observed in a sample for MHHs and FHHs, respectively; cells c) and d) denote the counterfactual expected productivity

$G = 1$ if the household head is male; $G = 0$ if the household head is female

$y_{m}$ = maize productivity indicator for MHH

$y_{f}$ = maize productivity indicator for FHHs

$BH_{m} \;{\text{and}}\; \, BH_{f}$ are the differences in productivity between the MHHs and FHHs, respectively, caused by unobserved factors

MHHsP and FHHsP denote the expected productivity effects of gender for those households randomly chosen from the MHHs and FHHs, respectively

If MHHs’ characteristics had had the same returns (coefficients) as FHHs’ characteristics returns (coefficients), then the effect of gender on MHHs’ maize productivity (MHHsP) could be given as the difference between Eqs. (3a) and (3c), as follows:

4 $$MHHsP = E(y_{m} |G = 1) - E(y_{f} |G = 1) = x_{m} (\beta_{m} - \beta_{f} )$$

Analogously, the effect of gender on FHHs’ maize productivity (FHHsP)—if their characteristics had the same coefficients as FHHs’ characteristics coefficient—is given by the difference between Eqs. (3d) and (3b), as follows:

5 $$FHHsP = E(y_{m} |G = 0) - E(y_{f} |G = 0) = x_{f} (\beta_{m} - \beta_{f} )$$

The MHHsP and FHHsP parameters give the expected maize productivity of a randomly chosen household from the MHHs and FHHs, respectively. Equations (4) and (5) are equivalent to the average treatment effect on the treated and on the untreated, respectively, in the impact evaluation literature, and to the coefficient effects in the literature on wage decomposition. The maize productivity of MHHs and FHHs may not be equal even if they have the same observed characteristics or even if they have the same returns to their respective observed characteristics. MHHs may have higher maize productivity than FHHs regardless of their observed characteristics due to other endogenous determinants of maize productivity (such as differences in access to extension services, credit, labor, education, and other productive inputs). This can be tested by taking the difference between Eqs. (3a) and (3d), and between those (3c) and (3b), as defined below:

6 $$BH_{m} = E(y_{m} |G = 1) - E(y_{m} |G = 0)$$

7 $$BH_{f} = E(y_{f} |G = 1) - E(y_{f} |G = 0)$$

Carter and Milon (2005) called Eqs. (6) and (7) as the base heterogeneity effect. The coefficients *β*_*m*_ and *β*_*f*_ are estimated from the Cobb–Douglas production function. However, because the *de facto* FHHs and *de jure* FHHs have fewer observations than the MHHs, we also estimate a Cobb–Douglas production function to check the robustness of the results. In doing this, the *de facto* FHHs and *de jure* FHHs are combined into FHHs.

## Results and discussion

### Descriptive statistics

Table 3 summarizes the statistics of the variables of our interest. Out of the total sampled households, the MHHs and FHHs represent 73% and 27%, respectively. Regarding FHHs, about 59% and 41% were *de jure* FHHs and *de facto* FHHs, respectively.

**Table 3:** Descriptive statistics and results from the test and mean differences by gender of household head

Variables description	Pooled sample [1]	MHHs [2]	All FHHs [3]	*De jure* FHHs [4]	*de facto* FHHs [5]	Test statistics	
						[2]--[3]	[4]--[5]
Outcome variable							
Total yield (ton/ha)	2.44	2.57	2.06	2.07	2.05	0.51***	0.02
Log [total yield (ton/ha)]	0.460	0.588	0.150	0.116	0.197	0.438***	− 0.081
Total value of product (Birr/ha)	7367.70	8105.7	5368.7	5498.7	5182.10	2737***	316.57
Log [total value of product (Birr/ha)]	8.345	8.465	8.022	7.993	8.063	0.443***	− 0.07
Independent variables							
Sex of the household head		0.73	0.27	0.16	0.11	0.46***	0.05
Age of the household head in years	42.61	42.20	43.72	45.40	41.20	− 1.52	4.20***
Education level of the household head in years	3.43	3.52	3.32	3.26	3.20	0.30	0.06
Size of household	6.18	6.66	4.90	4.53	5.44	1.76***	− 0.91
Number of children in the household (<15 years)	2.12	2.40	1.36	1.22	1.56	1.04***	− 0.34
Number of the males in the household (>15 years)	2.18	2.38	1.65	1.42	1. 98	0.73***	− 0.56**
Number of the females in the household (>15 years)	1.88	1.87	1.90	1.89	1.90	− 0.03	− 0.01
Male family labor work h/ha	179.14	179.08	179.28	178.78	179.99	− 0.20	− 1.21
Female family labor work h/ha	100.41	101.01	98.75	97.60	100.40	2.26	− 2.80
Total number of livestock owned by household in total livestock unit (TLU)	6.03	6.43	4.95	4.75	5.62	1.48***	− 0.87**
Number of oxen owned by the household	1.55	1.66	1.23	1.20	1.27	0.43***	− 0.07*
Total land holding of the household in hectares	1.58	1.70	1.24	1.30	1.16	0.46***	0.14**
Land used to grow maize in hectares	0.82	0.90	0.60	0.61	0.59	0.30***	0.02*
Amount of fertilizer applied (kg/ha)	90.20	98.50	83.20	80.60	86.80	15.30***	− 6.20*
Type of maize seed variety used (1 = improved)	0.65	0.68	0.55	0.52	0.53	0.13**	− 0.01
Access to credit service (1 = yes)	0.38	0.47	0.36	0.33	0.40	0.11**	− 0.07*
Contact with extension agent (1 = yes)	0.78	0.80	0.73	0.70	0.77	0.07*	− 0.07*
Distance from the market in km	10.88	9.80	11.27	10.62	14.37	− 1.47*	− 3.75**
Intercropping (1 = yes)	0.56	0.58	0.43	0.38	0.46	0.15**	0.08
Crop rotation (1 = yes)	0.60	0.64	0.50	0.50	0.48	0.14**	0.02
Agro ecology							
Lowland	0.57	0.57	0.55	0.58	0.50	0.02	0.08
Midland	0.38	0.38	0.37	0.35	0.42	0.01	− 0.07
Highland	0.05	0.04	0.07	0.07	0.08	− 0.03**	− 0.01
Number of observations	560	409	151	89	62		

*Source*: Own survey result (2018) ***, ** and * denote level of significance at 1%, 5%, and 10%, respectively. Birr is the Ethiopian official currency

The average yield of maize for sampled households was 2.44 tons/ha, which is lower than the national average of 2.66 tons/ha between 2007 and 2017. However, the average we found is twice as high as the national average yield in 2016, which was 1.2 tons/ha, and lower than the 2017 average of 3.67 tons/ha. On the other hand, our result is similar to the combined national average yield of 2016 and 2017, which was 2.43 tons/ha. In 2016, the Ethiopian agricultural sector was highly affected by the El Niño effect. As a result, the country’s national average crop yield, including maize, significantly dropped. As a response to the El Niño effect and because of maize’s wider environmental adaptability and higher yield potential than other cereal crops in Ethiopia (Abate et al. 2015), stakeholders involved in the crop sector played a major role in increasing maize production in 2016, particularly by converting plots of other crops to maize. However, production and yield remained lower in 2016 due to severe El Niño effects and related drought, disease, and pest attacks. In 2017, yield increased mainly due to an increase in maize production area coverage. Thus, our result might be the outcome of the shared effects from 2016 and 2017 production. In some parts of the Dawuro Zone, the influence of drought, diseases, and insect pests that were linked to El Niño effects were extended to the 2017 production season.

With respect to the gender of the household, Table 3 shows significant gender differences in maize yield between MHHs and FHHs. The difference is higher for *de jure* FHHs than *de facto* FHHs, though it is not significant. The average age of sampled households was 42.61 years with a higher average in FHHs. Compared to *de facto* FHHs; *de jure* FHHs are older in age. This could be linked to the migration of young married men in search of better income elsewhere.

For rural households that are unable to hire labor from the market, labor availability depends on the amount of family labor. The result of this study shows that the number of male family labor in the MHHs is significantly different from that in FHHs. That is, MHHs are larger, on average, by one person (2.38 versus 1.65 people). For FHHs, *de facto* FHHs have a higher number of family laborers than *de jure* FHHs. This result is consistent with the results of Djurfeldt et al. (2018) regarding six African countries.

Concerning family labor hours worked on the maize plot, male labor constituted a higher share of the total labor force than female. The total average share of female labor was about 36%. This is higher than the Ethiopian average of 29% reported by Palacios-López et al. (2017) but lower than the developing country average of 43% and much lower than the sub-Saharan African average of 50% (FAO 2011b). The main reason for this result may be related to the gendered labor division in agricultural production activities in Ethiopia. Another reason might be related to the dominant cultural views of labor in the study areas, which do not see farm work as a woman’s task. As a result, male family members may systematically over-report their labor contribution. In contrast, female family members may underreport their farm labor work because they consider their farm work as subservient to men. Moreover, the result might be linked to crop choice, given that women in the study area often engage in home garden activities while men work on the production of field crops such as maize. For field crops, women join men in later stages of production, such as weeding, harvesting, and collection. Land preparation and planting activities are carried out by ox-plowing, which requires physical labor. Women who are detached from ox-plowing tend to work much less on maize plots during the land preparation and planting period.

The average number of livestock (measured in Tropical Livestock Units) owned by the sample households was 6.03 TLU.^10^ MHHs own an average of 6.43 TLU, whereas FHHs own 4.95 TLU. This implies that an average MHH has a higher asset holding status than an average FHH. Among FHHs, *de facto* FHHs own more livestock than *de jure* FHHs. This indicates that the *de facto* FHHs had a higher asset holding status than *de jure* FHHs. This result is consistent with the other studies (i.e., FAO 2011a, b). Additionally, the average number of oxen owned by MHHs was higher than that of FHHs. This result is also in line with FAO (2011b) which notes that “women own fewer of the working animal needed in farming” (pp. 15). As for the ownership of oxen among FHHs, the *de facto* FHHs have more oxen than the *de jure* FHHs, which is likely related to their higher access to financial resources associated with remittances from and credit through their husband. The superior ownership of oxen by the *de facto* FHHs implies that they are more likely to use animal traction.

**Fig. 2:**
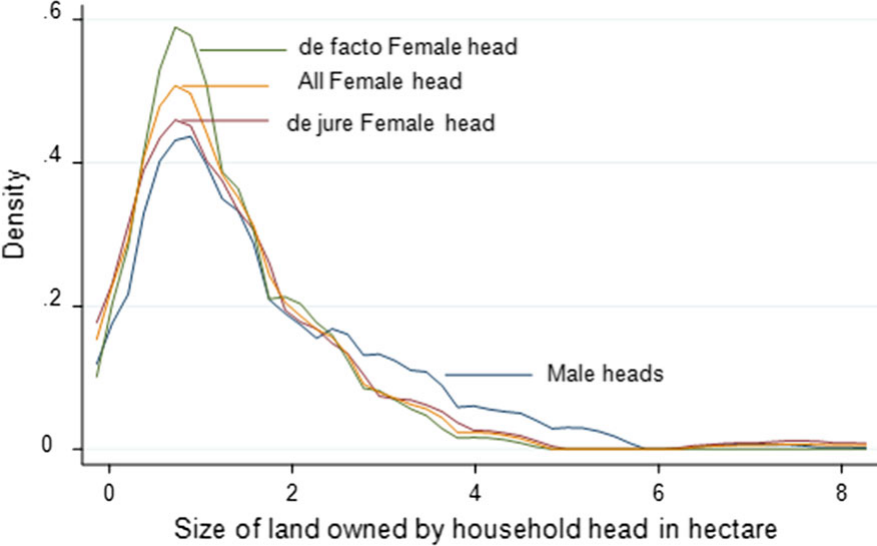
Distribution of landholding between MHHs and FHHs, *de facto* FHHs, and *de jure* FHHs — Kernel Density estimation Source: Author’s computation from the survey data (2018)

The total average landholding for sampled households was 1.58 ha, which is higher than the national and regional average holdings of 1.02 ha and 1.23 ha, respectively. The average land held by MHHs was significantly higher than that held by FHHs, which is in line with previous studies (i.e., FAO 2011b; Agarwal 2015). Regarding the landholding status among FHHs, the average land owned by the *de facto* FHHs is less than the *de jure* FHHs. This would suggest that some or many adult male partners of rural households leave home to seek non-farm job opportunities elsewhere because of a shortage of their own farmland for sustaining their livelihood. This result is in contrast with the result of Djurfeldt et al. (2018). The gender difference in landholding becomes more apparent when we compare the landholding distribution between MHHs and FHHs (Fig. 2). The distribution of landholding for FHHs is predominantly at the left of the MHHs distribution. The landholding distribution for *de facto* FHHs and *de jure* FHHs nearly overlap except the lower middle tail. The average land devoted to maize cultivation in the 2017 production season by MHHs and FHHs was 0.90 ha and 0.60 ha, respectively. However, among FHHs, *de jure* FHHs use more land area to cultivate maize. The key reason for this could be their larger landholdings compared to *de facto* FHHs.

Using modern inputs such as improved seeds and fertilizer are the most important factor for improving agricultural productivity.^11^ Our results show that about 65% of sampled households used the improved maize seeds; however, the adoption status was higher with MHHs at 68% than FHHs at 55%. The most commonly-used improved maize varieties reported by sampled households are BH540, BH660, and Pioneer hybrids. The amount of fertilizer applied to maize plots owned by MHHs was significantly higher than those owned by FHHs. These results are consistent with previous studies (i.e., World Bank 2009; FAO 2011b; Agarwal 2015). All of them note that women are less likely than men to use yield-enhancing inputs such as fertilizer and improved seeds. Among FHHs, *de jure* FHHs use less improved seeds and apply less fertilizer per hectare than *de facto* FHHs.

Households are also asked whether they accessed credit services during the maize production period. About 47% of MHHs and 36% of FHHs have access to credit from financial institutions.^12^ Among female heads, 40% of *de facto* FHHs and 33% of *de jure* FHHs have access to credit services. This implies that *de facto* FHHs still benefit from their husbands’ names and social network to access financial services in the community.

Extension services such as visits by and advice received from agricultural experts are designed to improve the farm productivity of rural households. However, various evidence (e.g., FAO 2011a; Ragasa et al. 2012) suggests that female heads tend to lag behind men in exploiting the benefits from extension services. The result of this paper shows that about 78% of sampled households have access to extension agents. Meanwhile, FHHs have less access to extension agents than MHHs. Among FHHs, *de facto* FHHs have more access to extension agents than *de jure* FHHs. This implies that an extension agent is more likely to visit *de facto* FHHs than *de jure* FHHs. The main reason for more access to extension services by *de facto* FHHs might be related to the name of the husband and his social networks.

### Model results

#### Factors affecting maize productivity

The estimates of the Cobb–Douglas production function are presented in Table 4. The coefficient of multiple determinations adjusted for degree of freedom implies that the variation in gross value of output per hectare associated with the factors of production specified in the model was 66.5% for the pooled sample, 63.4% for MHHs and 73.4% for FHHs (65.7% for *de facto* FHHs and 79.1% for *de jure* FHHs, respectively).

**Table 4:** Cobb–Douglas production function of factors affecting maize productivity by gender of the HH head

Variables	Pooled sample	MHHs	All FHHs	*de jure* FHHs	*de facto* FHHs
Log of land planted with maize/ha	0.686*** (0.046)	0.677*** (0.056)	0.705*** (0.088)	0.857*** (0.101)	0.530*** (0.184)
Log of fertilizer/ha	0.207*** (0.018)	0.200*** (0.021)	0.211*** (0.032)	0.172*** (0.039)	0.181*** (0.073)
Log of improved variety maize seed	0.533*** (0.093)	0.551*** (0.112)	0.410*** (0.163)	0.328** (0.212)	0.603*** (0.294)
Log of family male labor h/ha	0.187** (0.021)	0.143*** (0.054)	0.294*** (0.041)	0.357** (0.023)	0.272** (0.070)
Log of family female labor h/ha	0.031* * (0.060)	0.068* (0.072)	0.112** (0.156)	0.098** (0.076)	0.107** (0.080)
Log of number of oxen owned	0.207*** (0.127)	0.275** (0.155)	0.181** (0.221)	0.472* (0.279)	0.253* * (0.434)
Log of other livestock in TLU	0.076 (0.056)	0.060 (0.066)	0.145 (0.103)	− 0.013 (0.128)	0.299* (0.204)
Age of the household head in years	− 0.003 (0.003)	− 0.008** (0.003)	0.010** (0.005)	0.014** (0.006)	− 0.012 (0.015)
Education level of household head in years	0.009 (0.008)	0.010 (0.009)	0.018 (0.015)	− 0.000 (0.019)	0.053** (0.026)
Access to credit (1 = yes)	0.053 (0.060)	0.046 (0.073)	0.030 (0.107)	− 0.023 (0.132)	− 0.007 (0.201)
Contact with extension agent (1 = yes)	0.130* (0.078)	0.117 (0.096)	0.112 (0.129)	0.054 (0.146)	0.324 (0.301)
Distance from the market in km	− 0.005 (0.005)	− 0.007 (0.005)	− 0.002 (0.008)	− 0.003 (0.012)	− 0.026 (0.022)
Intercropping (1 = yes)	− 0.007 (0.058)	0.013 (0.070)	− 0.047 (0.102)	− 0.006 (0.133)	− 0.109 (0.178)
Crop rotation (1 = yes)	0.096 (0.057)	0.178** (0.070)	0.086 (0.096)	0.107 (0.121)	− 0.090 (0.192)
Agro− ecology (1 = lowland)	0.458*** (0.131)	0.465** (0.173)	0.452** (0.193)	− 0.079 (0.251)	0.741** (0.322)
Agro-ecology (1 = mid land)	0.285** (0.131)	0.251 (0.175)	0.243 (0.190)	− 0.080 (0.235)	0.530 (0.327)
Sex of household head dummy	0.010 (0.067)				
Constant	6.269*** (0.449)	5.802*** (0.625)	5.014*** (0.739)	7.063*** (0.962)	4.313*** (0.448)
F-ratio	65.96	45.12	26.80	21.83	8.21
Prob > F	0.000	0.000	0.000	0.000	0.000
R^2^	0.675	0.649	0.763	0.829	0.749
Adj R^2^	0.665	0.634	0.734	0.791	0.657
Number observations	560	409	151	89	62

*Source*: Own survey result (2018). ***, ** and * denote level of significance at 1%, 5% and 10%, respectively. Standard Errors are given in parentheses

The significant factors affecting the gross value of output per hectare for the pooled sample are farmland size, fertilizer, improved variety seed, family laborers, number of oxen, contact with extension agent, and agro-ecological variations. The coefficient for the gender dummy is not significantly different from zero, indicating that the sex of the household head does not have a significant impact on the production, process, at least in this model. This result is similar to the findings of Moock (1976), Njuki et al. (2006), Thapa (2008), and Aguilar et al. (2014).

In the pooled sample, farmland size had a positive and significant effect on the gross value of maize output per hectare. This result is in line with the results of Collier and Dercon (2014), Savastano and Scandizzo (2017), and Gollin (2018) but contrasts with the findings of Njuki et al. (2006), Oseni et al. (2015), Aguilar et al. (2014), and Mukasa and Salami (2015). According to Njuki et al. (2006), the inverse relationship between farmland size and productivity in Kenya is related to producers’ limited access to and relatively high cost of agricultural inputs. As farmers farm on a larger area of land, they are increasingly less capable of using a sufficient amount of inputs to maintain productivity. In their study on three African countries (Nigeria, Tanzania, and Uganda), Mukasa and Salami (2015) suggest that female farmers would have an advantage in terms of productivity over male farmers because they cultivate smaller farms, on average. Collier and Dercon (2014) and Gollin (2018) argue that small farms can be productive but not in the sense of technological productiveness but because of the imperfection in factor (e.g. labor) markets. Hence, the persistent emphasis on the inverse productivity relationship in the debate on large versus small-scale production is methodologically flawed. Meanwhile, in their study on Ethiopia, Savastano and Scandizzo (2017) identified a nonlinear significant U-shape relationship between farm size and productivity. They explain that, given a level of productivity, the relationship is direct for very small and large farms while inverse for middle-sized farms. Since our study focuses on smallholder farmers, the finding of a direct relationship between land size and maize productivity may be relevant to the study (on U-shape relationship).

The coefficients of fertilizer and improved seeds are also positive and significant. Given other inputs, a 10% increase in the use of improved maize seed would result in an increase in the value of gross output by 5.33% whereas a 10% rise in the use of inorganic fertilizer can increase the value of gross output by 2.07%. This result is in line with the result of Saito et al. (1994), Tiruneh et al. (2001), and Aguilar et al. (2014).

Both male and female family labor hours have a significant positive sign in pooled regression. Given other inputs, a 10% increase in the use of family male labor results in a 18.7% increase in the value of gross maize output. Other inputs, such as number of farm oxen and contact with extension agents, have a positive significant effect on the value of gross outputs.

The coefficients of agro-ecological variations of the maize plots show that when we go from highland to lowland agro-ecologies, the value of the gross output increases. This may be related to: (1) use of local maize varieties without knowing its appropriateness to specific agro-ecologies; and (2) the size of maize farmland and experiences of growing maize. In the study area, maize plots located in lowland agro-ecologies are the largest in size followed by midland and highland agro-ecologies, respectively. Moreover, farmers in highland agro-ecologies are more experienced in growing pre-annual crops than maize production. These factors may explain our result.

The estimated coefficients from separate regressions show that farmland size, fertilizer, improved seed, male and female family labor, and farm oxen had positive and significant effects on the value of gross output for both MHHs and FHHs. The effects of farmland size, fertilizer, and male and female family labor are higher for FHHs than MHHs. For instance, a 10% increase in the use of fertilizer on maize farms will increase the value of gross output by 2.11% for FHHs and 2% for MHHs. Likewise, a 10% increase in maize farm size will increase the value of its gross output by 7.05% for FHHs and 6.77% for MHHs. The result of the positive relationship between fertilizer and maize output is in line with the results of Tiruneh et al. (2001) that a 10% increase in the use of inorganic fertilizer resulted in a 1% increase in the value of gross output for both MHHs and FHHs in Ethiopia. In this study, a 10% increase in the use of improved maize seed would increase the value of gross output by 5.51% and 4.10% for MHHs and FHHs maize plots, respectively. This implies that the use of improved seed has more impact on the value of the gross output of MHHs farms than their female counterparts.

Similarly, the coefficients of farmland size, fertilizer, improved seed, farm oxen and family labor have positive and significant effects on the value of gross output for *de facto* FHHs and *de jure* FHHs, but the level of effects are different. For example, farmland size has more effect on the maize output of *de jure* FHHs than *de facto* FHHs while the use of fertilizer and improved seed has more effect on maize output of *de facto* FHHs. This indicates that the larger landholding status gives *de jure* FHHs an advantage in producing maize output than *de facto* FHHs. Conversely, *de facto* FHHs, who may have more financial resources from their husbands’ remittances, would be more capable than *de jure* FHHs of investing in and benefitting from the use of fertilizer and improved seed for maize production. Moreover, the education level of the head has a significant and positive effect on the value of maize output.

The coefficient of the age of household head has a significant effect on the value of gross output with a negative sign for MHHs and a positive sign for FHHs. This indicates that farm experience related to the age of household head tends to benefit FHHs but not MHHs. The negative relationship identified in this study between the age of MHHs and gross output is in line with the findings of Tiruneh et al. (2001) in Ethiopia, which suggest that a 10% increase in the age of MHHs will result in a 2.1% decrease in farm gross output.

Crop rotation, which is considered a good agricultural practice to increase productivity, tends to benefit MHHs more than their female counterparts. It has positive effects on the value of maize output for both types of households, but its impact on FHHs’ maize output is not significant.

#### Resource efficiency

Allocative efficiency can be determined by comparing the marginal value product (MVP) of a factor with its factor price (opportunity cost). However, for unpaid family labor, the shadow wage rate, which is the marginal value product of family labor, represents its opportunity cost (Abdulai and Regmi 2000; Sharma 2013). The MVP of a factor is the additional return from adding one more unit of that factor, holding all other factors constant. To suggest the efficient use of the resource for maize production, the ratio of the MVP of a factor and its price must be equal to one. If the ratio exceeds one, it implies that there is more scope for productivity by increasing the use of that factor. Conversely, if the ratio is less than one, it implies that increasing use of that factor will decrease productivity.

Table 5 shows the MVP and factor prices for a set of important variables related to production such as land, family labor, fertilizer, and improved seed. To avoid input price variations across the different villages, the opportunity cost of each factor was calculated based on median prices of respective factors in Ethiopian Birr (ETB).^13^ Since the study aimed to compare MVP with its factor price, for family labor inputs, we considered the actual wage rate of hired labor based on the assumption of perfect substitution between family and hired labor. However, in accordance with the social perceptions and physical requirements of farming activities in the study area, male laborers receive a higher wage rate than female laborers for the same amount of time they spend in the field. Henceforth, the median actual wage rates for male and female laborers on an hourly basis are 4.37 ETB and 3.75 ETB, respectively. The computed median price for a unit (kg) of fertilizer and improved maize seed is 13 ETB and 7 ETB, respectively. The government tax, which is used as rent in this paper, for a hectare of land is 350 ETB.^14^

**Table 5:** MVPs and factor prices

Factor	MHHs		All FHHs		*de facto* FHHs		*de jure* FHHs	
	MVP	Factor price	MVP	Factor price	MVP	Factor price	MVP	Factor price
Family male labor (h/season)	10.35	4.37	9.97	4.37	12.14	4.37	9.20	4.37
Family female labor (h/season)	7.53	3.75	8.90	3.75	8.276	3.75	7.723	3.75
Farmland (ha)	6611.07	350	5793.84	350	6416.20	350	4734.56	350
Inorganic Fertilizer (kg/ha)	33.31	13	25.28	13	23.27	13	19.26	13
Improved seed(kg/ha)	20.07	7	11.45	7	9.37	7	16.30	7

*Source*: Own survey result (2018)

The findings show that the MVP of all considered inputs is higher than its factor price across all categories of households. The MVP to its price ratios of farmland size, fertilizer, and improved seed are higher for MHHs than FHHs, which implies that there is more potential to increase productivity by increasing farmland size, use of fertilizer, and use of improved seed for MHHs than their female counterparts. Meanwhile, the ratio of the MVP of male and female family laborers to their wage rates is higher for FHHs than their male counterparts, indicating that FHHs could increase their productivity by using more family labor. Among FHHs, the ratio of the MVP to its price of farmland size, fertilizer, and male and female family labor are higher for *de jure* FHHs than *de facto* FHHs, indicating that there is more scope for productivity by increasing male and female family labor, fertilizer, and farmland size for *de facto* FHHs than *de jure* FHHs. Conversely, the ratio of the MVP of improved maize seed to its prices is higher for *de facto* FHHs than *de jure* FHHs. This implies that there is more potential to increase productivity by increasing the use of improved maize seed for *de facto* FHHs than *de jure* FHHs.

#### Gender difference in maize productivity

Maize productivity is defined here using the monetary value (in Ethiopian Birr) of self-reported output per hectare. A self-reported difference in maize productivity between MHHs and FHHs is 44.3% (Table 3). The result is lower than found in a study by Challa and Mahendran (2015), which is 66.76% in south-western Ethiopia. Furthermore, it is higher than found in studies by Tiruneh et al. (2001), which is 35% in central Ethiopia and Aguilar et al. (2014), which is, on average, 23.4% in the four main regions of Ethiopia. However, the productivity difference in southern regional states of Ethiopia as identified by Aguilar et al. (2014) accounts for 61.4%, which is higher than in other regions in the country. The above simple comparison presented in Table 3 cannot provide the treatment effects of gender on maize productivity unless we make any comparable group based on both observable and unobservable characteristics that affect maize productivity. Thus, we estimated the conditionally-expected maize productivity and treatment effects of gender using the estimated coefficients from the ESTER as indicated in Table 6.

**Table 6:** Average maize productivity, treatment and heterogeneity effects between MHHs and FHHs

Household type	Household type		Treatment effect
	MHHs	FHHs	
MHHs	(a) 8.465	(c) 7.860	0.605***
FHHs	(d) 8.445	(b) 8.022	0.423***
Heterogeneity effects	0.020	0.162	

*Source*: Own survey result (2018)

In general, one can say that a large proportion of FHHs would have higher maize productivity if they had the same observed resources and characteristics as MHHs. However, the difference between MHHs and FHHs would not be eliminated even if these observed differences were considered. That is, unobservable gender differences would have caused the female heads to have lower maize productivity than the male heads. Table 6 presents the actual and counterfactual differences in maize productivity between FHHs and MHHs. In Table 6, cell (a) represents the actual maize productivity for MHHs and cell (d) represents the counterfactual maize productivity conditions for FHHs. Cell (b) represents the actual maize productivity for FHHs, and cell (c) represents the counterfactual maize productivity conditions for MHHs. With these, we ask what the maize productivity level of FHHs would have been if the observed characteristics of FHHs had the same returns as that of MHHs, and vice versa. The difference between cells (a) and (b) in Table 6 provides us with an observed maize productivity gap between MHHs and FHHs, which is 44.3% in our case (Table 3). Our results show that this productivity gap would have been reduced by 2% if the FHHs had received the same level of returns on their productive resources as MHHs (as given by the difference between cells (a) and (d) of Table 6). That is, if the FHHs had received returns on their resources as equivalent to MHHs, their maize productivity increased by 42.3%, which nearly closes the existing gender productivity gap. This result indicates a marginalization of FHHs, which should be addressed by targeting this category of households for assistance. A policy approach, therefore, may aim at closing the gender inequality gap by providing FHHs equal access to productive resources such as extension, credit service, improved seed, and other important inputs in order to increase their maize productivity. Moreover, the policy should support FHHs so that returns to their productive resources are at least equivalent to those of MHHs.

**Fig. 3:**
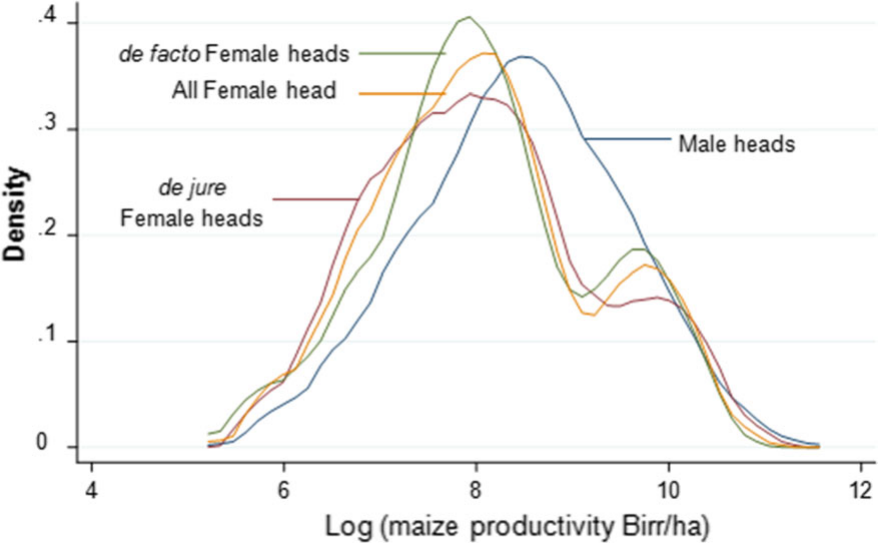
Maize productivity distribution between MHHs and FHHs, *de facto* FHHs and *de jure* FHHs—Kernel Density estimation Source: Authors computation from the survey data (2018)

The difference in maize productivity is also apparent when we compare the productivity distribution between MHHs and FHHs (Fig. 3). The distribution of productivity for FHHs is predominantly at the left of the MHHs distribution. A larger inequality is found in the middle of the productivity distribution. However, in the right-tail of the productivity distribution, the difference between MHHs and FHHs nearly overlaps. This indicates that at higher levels of productivity, returns on factors of production are similar for MHHs and FHHs.

## Conclusion and policy implications

This study reveals significant gender disparities in maize productivity in Ethiopia by applying the exogenous switching treatment regression approach for measuring production efficiency. In doing so, this study separately examined the impacts of the covariates on MHHs and FHHs. This procedure is different from many existing studies that only examine the intercept effect with the assumption of equal covariate impacts on productivity for MHHs and FHHs.

The study found that, on average, MHHs have a higher number of family members to provide labor, larger land size, and better financial access than FHHs, with statistical significance. Regarding differentiation within the FHHs, *de facto* FHHs are found to have a higher number of adult male family members to provide labor, a higher number of livestock units, and a smaller land size than *de jure* FHHs. The factors that significantly affect maize productivity for both types of household heads are the age of the head, farmland size, use of fertilizer, use of improved seed, access to male and female family labor, and farm oxen. Use of more farmland, fertilizer, and male and female family labor are found to affect FHHs more than MHHs. The result suggests that FHHs can benefit more from additional units of farmland, fertilizer, and male and female family labor than their male counterparts for maize production. Among FHHs, farmland size, male family labor, and farm oxen are found to benefit *de jure* FHHs more than *de facto* FHHs. In contrast, use of fertilizer, improved seed, and female family labor are found to benefit *de facto* FHHs more than *de jure* FHHs. The comparison of MVP and the factor price suggested that both MHHs and FHHs could increase productivity by using more land, family labor, fertilizer, and improved seed.

The study also found that, on average, the FHHs’ maize farmlands are 44.3% less productive in financial terms than the farmlands of their male counterparts. If the FHHs had received the same level of returns to their productive resources as MHHs, this productivity gap would have been reduced by 2.0%. In other words, their maize productivity would have increased by 42.3%. Moreover, the differences in productivity between MHHs and FHHs are not uniformly distributed across the productivity distribution. The difference increases as we approach the higher middle level of the distribution tails and then decreases as we move along the tail to the right. These findings suggest that, overall; the maize production sector favors males in the Dawuro Zone of southern Ethiopia. It therefore follows that the policy should aim at closing the gender gap by providing equal opportunities and access to productive resources for FHHs in order to increase their maize productivity. It is critical that the access to and provision of tailored extension services for FHHs be arranged in a technically more efficient manner to enhance their access to productive resources for raising maize productivity.

We recognize that one limitation of our study is that it relies on production data from a single crop to measure gender differences in agricultural productivity. We suggest that future studies on gender differences in agricultural production are based on aggregate crop production (production over different agricultural years, if possible). This addresses the recognition that gender gaps vary across various commodities as well as over agricultural production years. Second, where the amount of data allows, there is the need to carry out the exogenous switching treatment effect regression analysis done in this paper by disaggregating female-headed households into those that are *de facto* and those that are *de jure*.

## Footnotes

1 According to Doss (2018), “farmer” is defined as the owner, the manager, or the person providing the day-to- day labor for the plot or crop.

2 In this study, “household head” is defined as an individual who administers the household or an individual who is considered responsible for the other household members (Ragasa et al. 2012), including household farm land entitlement.

3 Kebele is the smallest administrative unit that makes up a woreda (district) in Ethiopia.

4 The ten woredas of Dawuro Zone are Essara, Kachi, Tocha Mari Mantsa, Maraka, Tarcha Zuria, Gena, Zaba Gazo, Loma, and Zisa. Thus, in the current administrative formation of Dawuro Zone, the representative sample farm households included in this study are from Loma, Zisa, Mareka, Kachi, Tarcha Zuria, and Essera woredas.

5 In Ethiopia, there are two crop production seasons: “Meher” and “Belg.” Meher is the main crop season between September and February and Belg is between March and August. Major crops, including maize, are grown during the Belg season and harvested during the Meher season (CSA 2016). In Dawuro Zone, particularly for maize production, land preparation activities commonly start in January. February to April is the planting season; May to August is the growth season depending up on maize varieties and agro-ecologies; and September to November is the harvesting period. December is the fallow period when no crop is grown on the maize plot.

6 Agricultural development (DA) agents in Ethiopia are also known as “extension agents” who graduated from the Agricultural Technical and Vocational Education Training (ATVET) College and are working at the kebele level. Three DA agents are assigned to each kebele to provide effective extension services for farmers in the areas of crop and livestock production and natural resource management. Agricultural extension implementation modality differs slightly from region to region in Ethiopia. Regional states also differ in how they supply agricultural inputs to farmers. In SNNP regional state, improved maize seeds and fertilizer are supplied to farmers through government extension agents (Leta et al. 2017). In each kebele, they supply these inputs to farmers without significant price variation. The prices of improved maize seeds and fertilizer across different kebeles are nearly uniform in a given production season.

7 In this study, family farming is defined as farming activities operated and managed by family members.

8 In this study, only the labor input of adult family members was considered. According to Palacios-López et al. (2017), the adult household members contributed at least 90% of all labor devoted to the crop sector in Ethiopia. The inclusion of child labor does not significantly affect the labor share by gender in the country. Moreover, the sampled households did not use any hired labor for maize production. Thus, our data do not include information on the labor input of hired labor.

9 Productivity is measured as the sum of the total value of maize product divided by the total area of land (in hectares) used to grow maize in the 2017/2018 production season.

10 Tropical Livestock Units are livestock numbers converted to a common unit (HarvestChoice 2015). Conversion factors are: cattle = 0.7, sheep = 0.1, goats = 0.1, pigs = 0.2 and chicken = 0.01.

11 This study considered only mineral fertilizers such as Urea, DAP and NPSB (Nitrogen, Phosphorous, Sulfur, and Boron-based) fertilizers. Previous studies such as Abate et al. (2015) note that potassium fertilizers are not considered important in Ethiopian agriculture, given the prevailing perception that Ethiopian soils are not deficient in this kind of mineral.

12 In the study area, the main financial institution providing loans for agricultural inputs, reported by sampled households, is the Omo Micro Finance. It provides loans to the active poor with good work habits, who are honest permanent residents of the village, and who have productive assets such as land for collateral for loaning. The loan amount for agricultural inputs from this institution depends on the loan cycle. In the first cycle, the loan amount is small but can be sufficient to purchase improved seeds and fertilizer for smallholder farmers. However, the amount can be increased in the second, third, fourth and fifth cycles, respectively, depending on the repayment history of the farmer (Kidane et al. 2004).

13 Note: 1 ETB = 0.036 USD during the data collection period. Factor prices are measured using ETB per respective unit of that factor.

14 The maize plots of all the sampled households are either own holding received from the government or inherited from parents but not rented from private sources.
